# The Perception of Deceptive Information Can Be Enhanced by Training That Removes Superficial Visual Information

**DOI:** 10.3389/fpsyg.2018.01132

**Published:** 2018-08-17

**Authors:** Donghyun Ryu, Bruce Abernethy, So Hyun Park, David L. Mann

**Affiliations:** ^1^Institute for the Psychology of Elite Performance, School of Sport, Health and Exercise Sciences, Bangor University, Bangor, United Kingdom; ^2^School of Human Movement and Nutrition Sciences, University of Queensland, Brisbane, QLD, Australia; ^3^School of Public Health, Li Ka Shing Faculty of Medicine, The University of Hong Kong, Pokfulam, Hong Kong; ^4^Te Huataki Waiora Faculty of Health, Sport and Human Performance, University of Waikato, Hamilton, New Zealand; ^5^Department of Human Movement Sciences, Amsterdam Movement Sciences and Institute of Brain and Behavior Amsterdam, Vrije Universiteit Amsterdam, Amsterdam, Netherlands

**Keywords:** deception, anticipation, perceptual training, interception, sport

## Abstract

The ability to detect deceptive intent within actions is a crucial element of skill across many tasks. Evidence suggests that deceptive actions may rely on the use of superficial visual information to hide the basic kinematic information which specifies the actor’s intent. The purpose of this study was to determine whether the ability of observers to anticipate deceptive actions could be enhanced by training which removes superficial visual information. Novice badminton players (*n* = 36) were allocated to one of three groups who performed perceptual training over 3 days, with the efficacy of training assessed using tests of anticipatory skill conducted at pre-test, post-test, and a 1-week retention test. During training, participants watched a series of non-deceptive badminton shots performed by actors, with the footage manipulated to display either (i) low spatial-frequency information only (*low-SF training group*; blurring to remove superficial information); (ii) high spatial-frequency information only (*high-SF training group*; an ‘edge detector’ to highlight superficial information); or (iii) normal vision (*normal-SF group*). Participants were asked to anticipate the direction of the shuttle when footage was occluded at the moment of racquet-shuttle contact. In the post-test, response accuracy (RA) when viewing deceptive trials was higher for the low-SF training group when compared to the normal-SF (control) training group (*p* = 0.005), with the difference retained in the retention test (*p* = 0.020). High-SF training resulted in greater performance at post-test (*p* = 0.038) but not retention (*p* = 0.956). The analysis of gaze provided some explanation for the findings, with the low-SF training group spending more time after training fixating on the location of racquet-shuttle contact than did the normal training group (*p* = 0.028). The findings demonstrate that training which conveys only the basic kinematic movements visible in low-SF information may be effective in learning to ‘see-through’ deceptive intent.

## Introduction

The ability to identify deceptive intent can be crucial in a variety of social contexts (Cañal-Bruland, 2017). For instance, during verbal communication it is important to be able to detect when others are lying (Ekman et al., 1999), or even when a person is dis-ingenuine in what they are saying (Vrij and Mann, 2004). Deceptive intent is also conveyed during *physical interactions* when observing the actions embodied within the movements of others (Jackson et al., 2006). This is particularly the case in a variety of sports, where deception is often used by athletes to fool their opponents into making an incorrect judgment about that athlete’s true action intentions (Cañal-Bruland and Schmidt, 2009). But while athletes may invest considerable time in learning to *perform* deceptive actions (e.g., a rugby side step, change-up baseball pitch, or head fake in basketball), it is also important for athletes to learn to ignore or ‘see through’ this deceptive intent to avoid errors, and to better anticipate the genuine action intentions of their opponent.

Runeson and Frykholm (1983) were the first to investigate and report the ability of observers to detect deceptive intentions when watching others perform a motor task. Participants in their seminal study watched point-light displays of actors who lifted boxes onto a table, and in a subsequent experiment, watched actors who in some cases attempted to deceive observers by pretending that the box they were lifting was heavier than it actually was. The results revealed that not only were the observers able to accurately estimate the weight of the box when the actors performed genuine actions, but that the observers were also successful in detecting when the actors were attempting to deceive them. Given that the point-light displays conveyed only very basic information about the underlying kinematic pattern of body movements of the actors, these results highlight that information available from the basic kinematic signature of the actor can be sufficient for even novice observers to perceive the genuine action intentions of both deceptive and non-deceptive actions.

The ability to ‘see through’ deceptive intent is a skill that can be learned seemingly as a result of domain-specific experience (Jackson et al., 2006; Cañal-Bruland, 2017). As evidence, Jackson et al. (2006) tested the anticipatory skill of skilled and novice rugby players who watched video footage of opponents performing deceptive and non-deceptive side-step running actions. The results revealed that, when attempting to anticipate the direction in which the opponent would run, the skilled players were less susceptible to deception, meaning that they were better able to ignore the deceptive intent and anticipate the true action intentions of the opponent. The implication of this finding is that skilled performers are characterized by their better ability to discriminate deceptive from non-deceptive actions (Cañal-Bruland and Schmidt, 2009; Sebanz and Shiffrar, 2009; Abernethy et al., 2010a,b; Cañal-Bruland et al., 2010), but also that the ability to detect deception may be a learned skill that could be enhanced as a result of training.

In an effort to guide improvements when training to perceive deception, it is important to gain an understanding of how deceptive information is most effectively conveyed. Crucially, there is good reason to believe that deceptive intent is conveyed at least in part by the detailed *non-kinematic* information available such as the gaze direction and facial expressions seen when observing an opponent’s action sequence. On the basis of Runeson and Frykholm (1983) finding that deception was unsuccessful when observers viewed a point-light display of a box-lifting the action, Abernethy et al. (2010a,b) examined the ability of badminton players to anticipate the direction of deceptive and non-deceptive badminton shots when viewing both video clips and point-light displays of the same shots. The results revealed that watching videos, the observers’ ability to discriminate deceptive from non-deceptive shots was worse than it was when watching the point-light displays. In other words, when watching the point light displays, the badminton players were less likely to be deceived than when watching the equivalent video clips. When watching video clips, a range of non-kinematic sources of information are available that are not seen when watching a point-light display, including information conveying contour, color, texture, and detail such as facial expressions and the direction of gaze. The clear implication from the findings from Abernethy et al.’s (2010a,b) studies is that deceptive intent can be conveyed largely via these *non-kinematic* sources of information. In contrast, the *kinematic* signature contains the specifying information that may be necessary for the anticipation of action outcomes, irrespective of whether deceptive intent is present or absent.

Given that deceptive intent is contained within non-kinematic information, a perceptual training approach that removes or degrades this non-kinematic information may hold promise as a means of improving the ability to anticipate deceptive actions. A considerable proportion of the non-kinematic sources likely to be useful for deception is contained within information that is highly detailed, meaning that clear vision would be required to resolve that information (e.g., facial expressions and gaze direction), whereas this is not necessarily the case for the more coarse kinematic information available from point-light displays. This means that information that *does* convey deception could be disambiguated from that which does not on the basis of the quality of the visual information relied on to convey it. An image, just like a sound, can be decomposed into component frequencies called *spatial frequencies*. When an image is blurred, the detailed high spatial frequency (SF) information is removed from the image so that only the low-SF information remains. Conversely, an edge-detecting ‘high-pass’ filter will produce an image of high-SF by removing the low frequency information. It is widely accepted that human observers prefer high SF information when making a conscious observation of an image (Harmon, 1973; DeValois and DeValois, 1990). Yet it appears that it is the low spatial frequency information that may be most useful for the perception of motor actions.

A small number of studies have demonstrated that an observer’s ability to make judgments about moving or changing stimuli can be enhanced by blurring the vision of the observer (e.g., di Lollo and Woods, 1981; Luria and Newacheck, 1992; Jackson et al., 2009; Mann et al., 2010c; Ryu et al., 2015, 2016). A possible explanation for each of these studies is that the blur aided the perception of movement by removing the high SF information which observers are consciously drawn to, leaving only the low SF information most useful for the perception of motion. When examining the anticipation of motor actions, Jackson et al. (2009) found that a high level of full-field blur *increased* the ability of tennis players to anticipate the direction of an opponent’s tennis serve. Similarly, Mann et al. (2010c) found that visual blur increased the capability of skilled cricket batters to verbally anticipate the direction of cricket balls bowled toward them. It was reasoned in those studies that the improvements in performance could have been attributable to the removal of high spatial frequency information, helping to draw attention toward the low-SF information most useful for predicting action outcomes. Therefore, a training approach that educates the attention of observers toward the low rather than high-SF information contained within an action sequence may be useful for increasing the observer’s ability to avoid deception, and to therefore better perceive the genuine action intentions of an opponent.

The aim of this study was to determine whether the ability to anticipate actions in the presence of deception could be enhanced by training that removes superficial (high-SF) visual information. To this end, participants watched a series of badminton shots, with the aim to anticipate the direction in which the player hit the shuttle. Following a pre-test of anticipatory skill, novice participants were split into one of three training groups who received feedback when anticipating the outcome of movement sequence seen in footage showing (i) low spatial frequencies only, (ii) high spatial frequencies only, or (iii) normal vision (control condition). Only non-deceptive actions were seen during training in an effort to minimize any training benefits accrued as a result of exposure to deceptive actions, and to train participants to focus on the relationship between genuine motor actions and their action outcomes. When observing veridical (non-deceptive) movements, we expected all three training groups to equally improve their anticipatory ability at post and retention test, because the true action intentions of the actor were evident during training irrespective of the type of visual information participants learned to rely on. In contrast, because deceptive information is likely to be conveyed more strongly by high-SF information, we expected that low-SF practice would result in the greatest improvement of all groups when anticipating *deceptive* movements in the post-test, because it would train observers to attend to the low-SF information more closely associated with the movement outcome. We expected the remaining two groups to perform more poorly, because they would rely on the high-SF information which is more likely to lead to susceptibility to deception.

## Materials and Methods

### Participants

Thirty-six participants (age *M* = 21.7 years, *SD* = 1.9) with limited experience playing badminton (*M* = 1.3 years, *SD* = 1.1) participated in this study. Participants were randomly assigned to one of three training groups: a *low-SF training* group (*n* = 12; playing experience *M* = 1.5 years, *SD* = 1.5); a *high-SF training* group (*n* = 12; playing experience *M* = 1.1 years, *SD* = 1.0); or a *normal-SF (control) training* group (*n* = 12; playing experience *M* = 1.4 years, *SD* = 0.9). The data for three participants were excluded from all analyses (one participant from each of the three groups, see ^∗∗∗^Dependent Variables and Data Analysis), leaving the data from 33 participants in the final analysis. Ethical approval was obtained from the University of Hong Kong Human Research Ethics Committee prior to testing, with informed consent obtained prior to the commencement of the experiment.

### Experimental Design and Procedures

#### Testing and Training Materials

##### Video clips

A series of video clips of badminton shots were used for the tests of anticipation and for the training footage. Five highly skilled players were recruited to be ‘actors’ for the purposes of recorded video footage. A digital video camera (Sony HDR-FX1 handicam) was used to record high-definition footage (1920 × 1080 pixel resolution) of strokes at 30 Hz, with the camera located at the center of the service court on the receiver’s side and at a height of 1.6 m. The actors stood at the intersection of the service and the doubles long service line and returned serves using only overhead strokes toward one of four landing positions on the court: front-left, back-left, front-right, and back-right. Only shots that landed within the playing court were included to be used as test stimuli. Players performed a series of *non-deceptive* and *deceptive* shots toward each of the four locations. When performing non-deceptive shots, actors attempted to hit the shuttle toward the intended direction without any deceptive intent. When performing deceptive shots, actors hit the shuttle toward the intended direction, but in doing so attempted to deceive an observer into thinking that the shuttle would be hit toward a different location on the court using any form of deception they would use in a regular match (kinematic and non-kinematic deception including gaze and head direction). A coach and scientist who worked regularly with the athletes within their sport institute were both present during filming to verify whether each shot matched the requirements of the condition and were representative of a shot that would be played in a match. Only those shots that matched those requirements were included as test films in the experiment. For each landing position, separate shots were recorded to convey deceptive intent in terms of depth and direction (e.g., for the front-left landing position, separate shots were recorded to deceive the observer into thinking that the shot was directed toward the back-left and the front-right sections of the court). The positions of the player and camera were chosen to simulate the respective locations on a court that a hitter may be expected to play ‘high-clear’ or ‘drop’ shots toward the back and front of the court respectively, and where a receiver would be required to move to intercept the four shots recorded.

**FIGURE 1:**
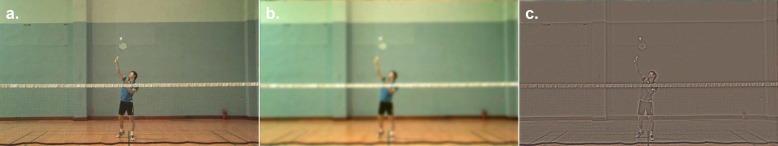
Demonstration of each of the three spatial frequency stimuli used in the training intervention; **(a)** Normal-SF information, **(b)** low-SF information only, and **(c)** high-SF information only.

Each of the clips was digitized, with the frames saved as individual high-definition bitmap images. These images were subsequently edited using Matlab software (version R2014b; Mathworks, Natick, MA, United States). Custom code was written in Matlab that resulted in two different manipulations of spatial frequency: (i) low spatial frequency (low-SF) images; and (ii) high spatial frequency (high-SF) images (see **Figure 1**). The normal images were the original (unfiltered) video images. When subtending the same visual angle as that experienced on-court, the normal images contained SF information ranging 0–22.7 cycles per degree (horizontally and vertically equating to 0–960 and 0–540 cycles per image respectively). HD video footage was chosen because standard definition video footage would have only contained spatial frequencies in the range of 0–12.1 cycles per degree. To generate the low and high-SF images, the normal bitmap images were respectively low- or high-pass filtered using a Gaussian filter with a cut-off of 4 cycles per degree. As a result, low-SF images were produced containing spatial frequencies ≈0–4 cycles per degree (**Figure 1b**), and high-SF images were produced containing spatial frequencies ≈4–22.7 cycles per degree (**Figure 1c**). To account for changes in brightness as a result of filtering, the brightness of both the low-SF and high-SF stimuli was matched to that of the original image. Each series of images was reconstructed into an HD video (1280 × 720 pixel resolution) using Sony Vegas Pro software (Version 13; Sony Creative Software, Middleton, WI, United States).

##### Test of anticipation

A total of 96 different video clips were used for the test of anticipation. To create the test, a selection of 32 video clips were chosen (8 deceptive and 8 non-deceptive from each of two actors; e.g., Mann et al., 2014), with each clip presented three times, but differing according to the moment of occlusion, either (i) one frame before contact between racquet and shuttle, (ii) at the moment of contact, or (iii) one frame after contact. The three occlusion times were chosen on the basis of pilot testing performed to establish occlusion point(s) at which pre-test performance would be above chance guessing levels but below the ceiling level. For each clip, participants were required to anticipate the landing position of the shuttle by pressing a button on a keyboard corresponding to one of the four landing positions. The order of trials was randomized. The test was conducted during the pre-test, post-test, and retention-test to assess the efficacy of the interventions.

##### Training material

A total of 360 video clips (all non-deceptive) were used for the training intervention. A set of 60 original clips (12 clips from each of the five performers) were occluded at each of the three occlusion times used for the test clips (i.e., 1 frame before, at contact, 1 frame after shuttle-racquet contact), with these 180 video clips shown two times across 4 different training sessions (i.e., a set of 90 video clips in each session, with all the clips randomized in each session). All five actors were shown during training to introduce novelty and minimize boredom. To provide explicit feedback about the direction of the shuttle during the training intervention, a replay of the clip was created that was edited to end 20 frames after the shuttle disappeared from the field of view. Moreover, feedback clips contained a schematic of a court overlaid on the upper-right hand corner of the screen, with the correct landing location marked with a red dot.

##### Eye movement registration system

An Eyelink II (SR Research Ltd., Mississauga, ON, Canada) was used at 250 Hz to check whether the eye movements of participants changed as a result of the different training interventions. The system was calibrated by asking participants to sequentially direct their gaze toward each of nine targets in a screen-based reference grid, and then validated in the same manner (acceptable error to <0.5°). Calibration was repeated if the error at any given point was >1°, or if the average error for all points was >0.5°. Eye movement data were analyzed using Data Viewer software (SR Research Ltd.).

#### Procedures

The experiment was conducted in four phases: a pre-test; intervention phase; post-test; and retention test. Participants were randomly assigned to one of three training groups: a *low-SF training* group; a *high-SF training* group; or a *normal-SF (control) training* group. Testing for each participant took 4 days in total, with the intervention taking place over three consecutive days. As a result, the pre-test and 1st training session were held on the 1st day, the 2nd and 3rd training sessions on the 2nd day, and the final training session plus post-test were held on the 3rd day. The retention test was scheduled 1 week after the post-test.

##### Pre-test

Participants sat with their head 60 cm from the Eyelink II display monitor (subtending a visual angle of 46.5° × 34.6°; screen size: 516 × 373 mm). Following the fitting and calibration of the gaze-registration system, an experimenter informed the participants of their task. Specifically, they were told they would see a series of video clips, each containing a badminton shot, and at the conclusion of each clip participants were required to predict as quickly and as accurately as possible in which quarter of the court that the shuttle would have landed, and to respond by pressing the corresponding button on a keyboard. Prior to testing, participants were given 12 practice trials to familiarize themselves with the test procedure. Then, they completed 96 test-trials which took approximately 30 min to complete.

##### Training intervention

The training intervention consisted of four training sessions of 90 video clips divided over three consecutive days. Just as it was for the test of anticipation, the task for participants during training was to predict for each clip the quarter of the court in which the shuttle would have landed. After watching each video clip, and recording their anticipated direction of the shuttle, 1 s of blank video was shown before the full (un-occluded) video clip was shown. The low-SF and high-SF training groups watched all clips with low and high spatial-frequency footage respectively, including the unoccluded feedback clips. The normal-SF training group watched the video clips with un-manipulated normal video footage. Each training session took approximately 30 min to complete.

##### Post-test and retention test

In the post and retention tests, participants were required to anticipate the shuttle direction for the same set of 96 clips shown in the pre-test, with the order of presentation of the clips following a different randomized order in each test.

### Dependent Variables and Data Analysis

#### Performance Data

Response accuracy (RA) and response time (RT) were calculated to evaluate performance in the pre-, post-, and retention-tests. RA was calculated as the percentage of trials in which the predicted landing position matched the actual position of the shuttle, and RT was the mean time (in ms) that elapsed from the moment the clip occluded to the time the participant’s keyboard response was registered. The raw data were initially screened, with one participant from the normal-SF training group excluded from all analyses because the participant, despite instructions, failed to respond at all in many clips, and as a result demonstrated consistently low RA across all the tests (lower than 2 SD below the mean). Moreover, one session of data from one participant in the low-SF training group, and one participant from the high-SF training group failed to save as a result of a technical issue, therefore the data from those two participants were also excluded from all analyses. In total, data from 33 participants were analyzed.

#### Gaze Behavior Data

First, to determine whether the duration of the visual fixations changed as a result of the training intervention, the *mean fixation duration* (in ms) was calculated for each trial by averaging the duration of all fixations in that trial. Second, to check whether the breadth of the search changed as a result of training, the *mean saccadic amplitude* (in degrees of visual angle) was determined by calculating the average angular subtense of all saccades in each trial. Finally, to assess whether the training altered the spatial locations toward which participants directed their fixations, the distribution of gaze across eight distinct areas of interest (AoI) was assessed for each trial by calculating the *percentage of total viewing time* spent viewing each of the eight areas. The eight AoIs chosen on the basis of pilot testing were: (i) shuttle, (ii) racquet, (iii) arm, (iv) hand and wrist, (v) shoulder, (vi) head, (vii) torso, and (viii) location of (racquet-shuttle) contact (to account for situations in which gaze moved toward this location in advance of the moment of contact). For the purposes of analysis, we placed boxes frame-by-frame around each of the AoIs to facilitate automatic coding of the location of gaze. That is, the Data Viewer software used the frame-by-frame boxes to determine the incidence and duration of fixations in each of the eight AoIs.

#### Statistical Analyses

In accordance with our aim to determine whether perceptual training would improve the ability to perceive action outcomes in the presence of deceptive intent, our analysis focuses on changes in RA and RT when viewing *deceptive* trials. We also report separately the findings for the non-deceptive trials to check whether the training also altered the ability to perceive actions in the absence of deceptive intent. The dependent variables measuring *RA* and *RT* were analyzed using separate 3 (Training group: normal-SF training, low-SF training, high-SF training) × 3 (Test occasion: pre-test, post-test, retention test) analyses of variance (ANOVAs) with repeated measures on the last factor. Gaze behavior data for the *mean fixation duration* and *mean saccadic amplitude* were analyzed using separate 3 (Training group) × 3 (Test occasion) ANOVAs with repeated measures on the last factor. The distribution of fixations toward the 8 AoIs (*percentage of viewing time*) were subject to a 3 (Training group) × 3 (Test occasion) × 8 (AoI) ANOVA with repeated measures on the last two factors. Further, the results for RA and RT collected during the training intervention were subject to a 3 (Training group) × 4 (Training session: first, second, third, fourth) ANOVA with repeated measures on the second factor to check for changes during training. Gaze data were not collected during training. Significant effects were further investigated using follow-up ANOVAs or planned comparison pairwise *t*-tests with Bonferroni correction where appropriate. Effect sizes were reported as partial eta-squared values or Cohen’s *d* (Cohen, 1988), and a Greenhouse–Geisser correction was applied to the degrees of freedom when the assumption of sphericity was violated. Statistical testing was performed in SPSS with the alpha level for all comparisons set to *p* = 0.05.

## Results

### Changes in Performance as a Result of Training

#### Deceptive Trials

**FIGURE 2:**
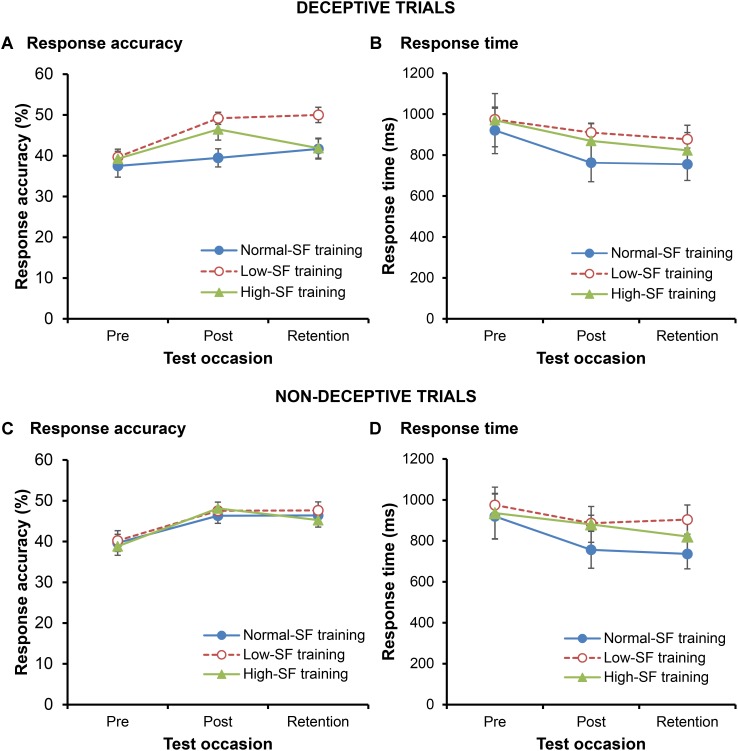
Mean response accuracy and response time for deceptive **(A,B)** and non-deceptive **(C,D)** trials. Error bars indicate the standard error of the mean.

A borderline interaction – with large effect size – between training group and test-time [training group × test occasion, *F*(4,60) = 2.12, *p* = 0.09, $\eta_{\text{p}}^{2}$ = 0.124] suggested that changes in the RA of the participants differed according to their type of training [main effect for training group, *F*(2,30) = 3.65, *p* = 0.038, $\eta_{\text{p}}^{2}$ = 0.195; main effect for test occasion, *F*(2,60) = 10.18, *p* < 0.001, $\eta_{\text{p}}^{2}$ = 0.253]. Specifically, **Figure 2A** shows that the low-SF training group significantly increased their performance from pre- to post-test (*p* = 0.008, *d* = 1.53), and that their enhanced performance at post-test was retained in the retention test 1-week later (*p* = 0.745, *d* = 0.13). While the high-SF training group also increased their performance from pre- to post-test (*p* = 0.023, *d* = 0.92), it is doubtful that this was retained when comparing performance in the post and retention tests (*p* = 0.075, *d* = 0.52). In contrast, there was no change in performance for the normal-SF training group either from pre- to post-test (*p* = 0.53, *d* = 0.23) or from post- to retention-test (*p* = 0.355, *d* = 0.27). In support, the RA of the low-SF training group was greater than that of the normal-SF training group at both post-test (*p* = 0.005, *d* = 1.46) and retention test (*p* = 0.02, *d* = 1.08), and it was also higher than the high-SF training group at retention (*p* = 0.023, *d* = 1.06). The high-SF training group recorded higher RA than the normal-SF training group only in the post-test (*p* = 0.038, *d* = 0.83) and not at retention (*p* = 0.956, *d* = 0.02).

The differences in the performance of the groups following training could not be explained on the basis of changes in RT (**Figure 2B**). RTs for all three groups decreased following training, both in the post-test (*p* = 0.045, *d* = 0.33) and retention test (*p* = 0.022, *d* = 0.40) when compared to the pre-test [main effect for test occasion, *F*(1.33,39.94) = 4.70, *p* = 0.027, $\eta_{\text{p}}^{2}$ = 0.135]. There was no change in RT from post-test to retention test (*p* = 0.383, *d* = 0.08). However, the rate of change in RT as a result of training did not differ between the three training groups, with no significant interaction between training group and test occasion [*F*(2.66,39.94) = 0.47, *p* = 0.685, $\eta_{\text{p}}^{2}$ = 0.03; no main effect for training group, *F*(2,30) = 0.56, *p* = 0.577, $\eta_{\text{p}}^{2}$ = 0.036].

#### Non-deceptive Trials

In the non-deceptive trials, RA increased as a result of training [main effect for test occasion, *F*(2,60) = 17.68, *p* < 0.001, $\eta_{\text{p}}^{2}$ = 0.371], however, the degree of improvement did not differ between the three different training groups [**Figure 2C**; no group × test occasion interaction, *F*(4,60) = 0.28, *p* = 0.892, $\eta_{\text{p}}^{2}$ = 0.018; no main effect for training group, *F*(2,30) = 0.17, *p* = 0.85, $\eta_{\text{p}}^{2}$ = 0.011]. RA significantly increased from pre-test to post-test (*p* < 0.001, *d* = 1.19), and remained higher in the retention test than it was at pre-test (*p* < 0.001, *d* = 0.94), with no significant change from post-test to retention test (*p* = 0.421, *d* = 0.15).

Again, the improvements in RA as a result of training were accompanied by decreases in RTs [main effect for test occasion, *F*(1.27,37.94) = 6.41, *p* = 0.011, $\eta_{\text{p}}^{2}$ = 0.176] that did not differ according to the training intervention [no group × test occasion interaction, *F*(2.53,37.94) = 0.27, *p* = 0.816, $\eta_{\text{p}}^{2}$ = 0.018; no main effect for training group, *F*(1,20) = 0.09, *p* = 0.767, $\eta_{\text{p}}^{2}$ = 0.004]. RTs decreased following training (*p* = 0.027, *d* = 0.34), and remained lower in the retention test when compared to the pre-test (*p* = 0.008, *d* = 0.43). There was no difference in RTs between post-test and retention test (*p* = 0.154, *d* = 0.11).

### Changes in Gaze Behavior as a Result of Training

**FIGURE 3:**
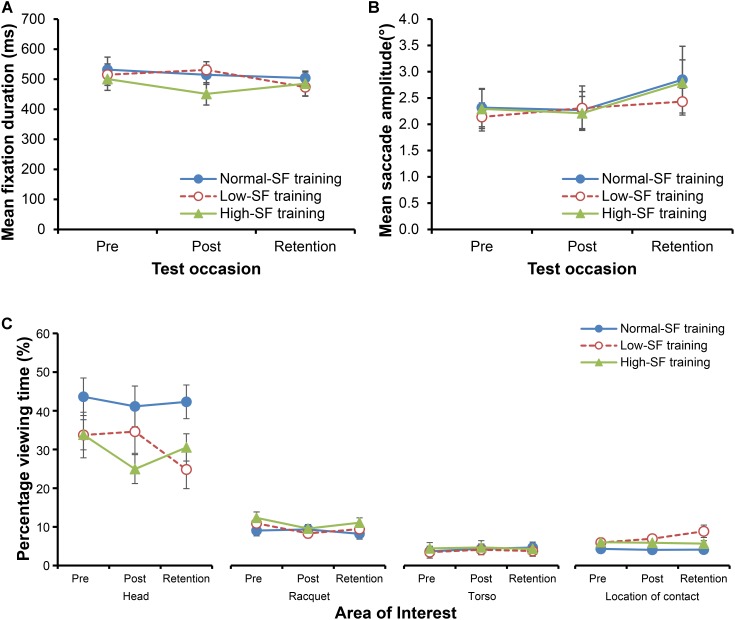
Mean fixation duration **(A)** and mean saccadic amplitude **(B)**. Percentage of total viewing time toward each of four key AoIs for each group **(C)**. To reduce complexity, only the four most frequently fixated AoIs are shown: head, racquet, torso, and location of contact. Error bars indicate the standard error of the mean.

There was no change in the duration of the fixations as a result of training for any of the three groups [**Figure 3A**; no main effect for training group, *F*(2,30) = 0.40, *p* = 0.673, $\eta_{\text{p}}^{2}$ = 0.026; no main effect for test occasion, *F*(1.52,45.49) = 1.87, *p* = 0.173, $\eta_{\text{p}}^{2}$ = 0.059; no interaction between group and test occasion, *F*(3.03,45.49) = 1.70, *p* = 0.18, $\eta_{\text{p}}^{2}$ = 0.102]. Similarly, there was no influence of the type of training on the change in the breadth of the search (**Figure 3B**). There was a borderline change in the mean saccadic amplitude across test occasions [main effect for test occasion, *F*(2,60) = 2.98, *p* = 0.058, $\eta_{\text{p}}^{2}$ = 0.09], though primarily because there was a tendency for larger saccades in the retention test when compared to the pre (*p* = 0.036, *d* = 0.33) and post-tests (*p* = 0.066, *d* = 0.32). Crucially, any changes between test occasions did not differ according to the type of training performed by the participants [no interaction between group and test occasion, *F*(4,60) = 0.27, *p* = 0.893, $\eta_{\text{p}}^{2}$ = 0.02; no main effect for training group, *F*(2,30) = 0.08, *p* = 0.921, $\eta_{\text{p}}^{2}$ = 0.005].

The analysis of the percentage of total viewing time that was directed toward each of the 8 AoIs revealed a significant main effect for area of interest, *F*(1.33,39.78) = 147.74, *p* < 0.001, $\eta_{\text{p}}^{2}$ = 0.831. Pairwise comparisons revealed that most time was spent with gaze directed toward the head of the opponent, followed by their racquet, torso, and the location of contact (**Figure 3C**). The interaction between the AoI and training group was close to significance [*F*(2.66,39.78) = 2.61, *p* = 0.071, $\eta_{\text{p}}^{2}$ = 0.148], as was the three way AoIs × training group × test occasion interaction [*F*(4.96,74.38) = 1.93, *p* = 0.099, $\eta_{\text{p}}^{2}$ = 0.114]. Separate two-way ANOVAs on each key area of interest were conducted. A significant training group × test occasion interaction was found for the time spent viewing the location of racquet-shuttle contact [*F*(3.32,49.79) = 3.49, *p* = 0.019, $\eta_{\text{p}}^{2}$ = 0.189], with the low-SF training group spending more time than the normal-SF training group fixating on the location of racquet-shuttle contact both in the post-test (*p* = 0.028, *d* = 0.87), and the retention test (*p* = 0.007, *d* = 1.09), but not in the pre-test (*p* = 0.202, *d* = 0.51). Moreover, there was a borderline interaction between training group and test occasion for the percentage of time spent viewing the *head* of the opponent [*F*(4,60) = 2.12, *p* = 0.09, $\eta_{\text{p}}^{2}$ = 0.124]. When compared to the normal SF-group, the low-SF group spent significantly less time viewing the head at retention test (*p* = 0.007, *d* = 1.13), but not at pre-test (*p* = 0.167, *d* = 0.55) or at post-test (*p* = 0.357, *d* = 0.36). In contrast, the high-SF group spent less time than the normal-SF group viewing the head at post-test (*p* = 0.027, *d* = 1.07), but not at pre-test (*p* = 0.17, *d* = 0.67) or at retention test (*p* = 0.63, *d* = 0.90).

### Performance During Training

**FIGURE 4:**
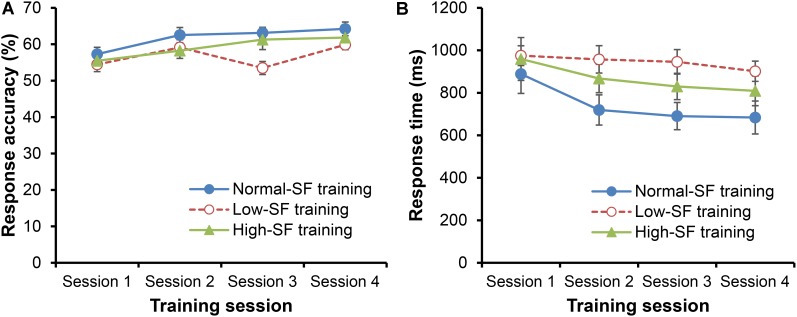
Mean response accuracy **(A)** and response time **(B)** during training intervention. Error bars indicate the standard error of the mean.

Response accuracy progressively increased during the training intervention [**Figure 4A**; main effect for training session, *F*(3,90) = 11.95, *p* < 0.001, $\eta_{\text{p}}^{2}$ = 0.285]. RA improved significantly from Session 1 to 2 (*p* < 0.05, *d* = 0.62), did not change from Session 2 to 3 (*p* = 0.582, *d* = 0.09), and ultimately improved again from Session 3 to 4 (*p* = 0.004, *d* = 0.38). There was also a significant interaction between training group and training session [*F*(6,90) = 2.46, *p* = 0.03, $\eta_{\text{p}}^{2}$ = 0.141; no main effect for training group, *F*(2,30) = 2.29, *p* = 0.119, $\eta_{\text{p}}^{2}$ = 0.132]. The interaction was seemingly due to the low-SF group unexpectedly performing worse than the other groups in Session 3 (*p*s < 0.018, *d*s > 0.96), but not in any of the other sessions (*p*s > 0.082, *d*s < 0.76).

The RT decreased for all training groups during training [**Figure 4B**; main effect for training session, *F*(3,90) = 7.73, *p* < 0.001, $\eta_{\text{p}}^{2}$ = 0.205]. However, the rate of change in RT did not differ according to the training group [no training group × training session interaction, *F*(6,90) = 0.99, *p* = 0.437, $\eta_{\text{p}}^{2}$ = 0.062; no main effect for training group, *F*(2,30) = 2.35, *p* = 0.113, $\eta_{\text{p}}^{2}$ = 0.136].

## Discussion

The purpose of this study was to determine whether the ability to anticipate deceptive actions could be enhanced by training that removes superficial visual information. Based on the idea that deceptive intent is conveyed at least in part via superficial (high-SF) information, we hypothesized that low-SF training would educate observers to attend to low rather than high-SF information, and ultimately lead to significant improvements in the ability to anticipate deceptive actions. The findings revealed that a low-SF group who trained viewing only low-SF information were the only training group to improve and retain their ability to anticipate the outcomes of deceptive actions at a level consistently above that of the control group. The high-SF group who viewed only high-SF information improved their performance from pre to post-test, but this improvement was not retained when tested in a 1-week retention test. Moreover, there was some suggestion that the training effect found for the low-SF group could be explained at least in part by a change in visual search behavior, with low-SF training leading to less time spent directing gaze toward high-SF information such as the opponent’s face, and more time spent viewing other areas such as the location of racquet-shuttle contact. Ultimately, the results are particularly striking in that the low-SF training led to retained improvements in the ability to anticipate *deceptive* actions, even though participants viewed only veridical (*non-deceptive*) actions during training.

The superior performance of the low-SF group provides further support for the idea that a substantial amount of the deceptive intent is conveyed during motor actions via high-SF information that may distract observers from the low-SF information that seems to be most useful for anticipation. Abernethy et al. (2010a,b) reported a decrease in prediction errors when observers anticipated deceptive actions while watching a *point-light display* rather than video footage of the same action. This result suggests that deceptive intent is contained within high-SF information, and that very simple (low-SF) kinematic information is sufficient for effective anticipation. Further support for the usefulness of low-SF information was provided by Jackson et al. (2009) and Mann et al. (2010c), who each demonstrated that the anticipatory judgments of athletes improved in some cases in the presence of blur. In the present study, we exploited those findings to hypothesize and show that training which taught observers to attend to low-SF information would improve the ability to ‘see-through’ deceptive intent. The superior performance of the low-SF, when compared to the normal-SF group, supports the idea that humans are distracted by high-SF information, and that athletes may not naturally attune directly to the low-SF information that seems to be most useful for anticipation. The findings highlight the need for observers to attend wherever possible to the coarse (low-SF) kinematic information which specifies genuine action outcomes, rather than attending to non-specifying information which is designed to fool or distract the observer.

The low-SF training group viewed low-SF information during training, yet improved and retained their anticipatory skill when tested viewing ‘normal-SF’ actions that contained both low and high-SF information. Even if the low-SF training was successful in training observers to make use of low-SF information when generating anticipatory judgments, it remained entirely possible that the high-SF information, when made available again in the post and retention-tests, could have been so pervasive that it would have distracted observers from the low-SF information they had learned to use. However, this was not the case. The improvement in anticipatory ability found as a result of training was retained even when viewing normal-SF information in the post-test, showing that the attunement to low-SF information learned during training ‘transferred’ to the more typical scenario when both low and high-SF information were available.

One of the most surprising outcomes of this study is that observers do not necessarily need to view deceptive actions in order to improve their ability to perceive them, but rather, that the anticipation of deceptive actions can be improved by an intervention which presumably trains observers to rely on the most useful information for anticipating action outcomes. Our study was designed in such a way that observers viewed only non-deceptive actions during training, a choice that was made in order to disambiguate any confounding influence of improved performance that might have been possible if participants became familiar with the deceptive actions. Instead, because deceptive actions were not seen during training, the findings provide some reassurance that the improvements seen when anticipating deceptive actions are the result of a fundamental change in the way that the participants in the low-SF group perceived the actions. An advantage for low-SF training was not found when viewing non-deceptive actions, with the improvement in anticipatory performance for the low-SF group being indistinguishable from that of the high and normal-SF groups when viewing non-deceptive actions in the post-tests. It may have been that the high-SF information that was available when viewing non-deceptive actions did not conflict with the low-SF information which specified the action outcome, and therefore there was no performance disadvantage for the high and normal-SF groups. Yet, when deceptive actions were viewed, it was only the low-SF group who improved and retained their ability to perceive action outcomes beyond that possible for the control group. In that case, their reliance on the highly specifying low-SF information may have made them less-susceptible to the high-SF information that conveys deceptive intent (Abernethy et al., 2010a,b).

The results from the analysis of gaze behavior provide some support for the idea that low-SF training leads to a fundamental change in the way that observers view and anticipate actions. While there was no change in the dynamics or extent of the visual search as a result of low-SF training (see also Ryu et al., 2016), there was evidence to show that the training altered *where* participants directed their gaze. In particular, as a result of training, participants in the low-SF training group decreased the proportion of time they spent viewing the face of their opponent, and increased the proportion of time spent viewing the anticipated location of racquet-shuttle contact. Given that the head is unlikely to be part of the kinematic chain responsible for producing a badminton shot (Abernethy and Russell, 1987), then it stands to reason that information from that location is unlikely to be particularly useful when predicting the outcome of an action (unless the opponent consistently directs their gaze toward the likely direction of the shuttle; Mareschal et al., 2013; Weigelt et al., 2017). Moreover, the information available from the opponent’s face can be very compelling and attract attention, often helping the actor to successfully fool or deceive an observer, for instance in the use of head fakes in basketball and soccer (Kunde et al., 2011). Because facial features were not clearly visible when blur was applied during low-SF training (see **Figure 1**), it may be that participants learned to ignore the opponent’s head/face, and instead focused their attention toward other more specifying areas of the visual array. Given that the most specifying kinematic information occurs late in the opponent’s action, the location of gaze late in the action is crucial. In interceptive tasks, skilled tennis players have been shown to reliably direct their gaze toward the anticipated point of racquet-ball contact immediately before contact (Williams et al., 2002), and in sports such as baseball and cricket, batters direct their gaze toward the anticipated location from which the pitcher/bowler will release the ball (McRobert et al., 2009; Mann et al., 2013; Sarpeshkar et al., 2017). It appears that the participants in the low-SF training group spent more time directing gaze toward the location of racquet-shuttle contact, and less time being distracted by information from their opponent’s face.

The results for the high-SF training group were surprising and are also worthy of further consideration. First, the high-SF group experienced a significant improvement in performance from pre to post-test when observing both deceptive and non-deceptive actions. One possible explanation for the improvement in the deceptive trials is that the deceptive intent conveyed via the high-SF information during the pre/post/retention tests might not have been entirely deceptive. That is to say, the high-SF information presented during a deceptive action might not *fully* replicate the high-SF information presented in the non-deceptive action the actor was seeking to replicate/convey. If that were the case, and the high-SF group did during training improve their ability to make judgments on the basis of high-SF information, then it may be that the observers were better able to perceive the attempted deception and to respond accordingly. An alternative explanation could be that as a result of the training, the high-SF group might have improved their ability to discriminate low from high-SF information, and then were able to rely more heavily on the low-SF information during the tests. The second finding of interest is that the improvement in RA from pre to post-test found for the high-SF training group had disappeared only 1 week later when tested at retention. The failure to retain improvements in performance following training is often attributed to the learned skills being acquired in an explicit rather than implicit manner (Maxwell et al., 2001; Masters and Maxwell, 2004). That is to say, if the skill is learned using an explicit approach, during which the learner accumulates declarative knowledge about how they should perform the skill, then the learned skill is more likely to be ‘forgotten’ over time (Allen and Reber, 1980). It may be that the high-SF training group acquired their skill in a more explicit manner than the low-SF group. The very pervasive nature of the detailed high-SF information may have led the high-SF group to focus explicitly toward specific information in the action sequence and to develop conscious rules about the meaningfulness of the high-SF information. In contrast, the low-SF group did not have access to this detailed information during training and may have instead focused on the coarse kinematic information that humans would typically rely on when judging the movements of others (Troje, 2002). Similarly, it could be that the overt nature of the high-SF information distracted the observer from relying on the low-SF information that better specifies the action outcome, increasing the likelihood that information was processed in a bottom–up rather than top–down fashion (Corbetta and Shulman, 2002; Carrasco, 2011). If true, Attention Control Theory (Eysenck et al., 2007) would suggest that, under anxiety, observers viewing deceptive actions should become more readily deceived, because top–down processing would be impaired and so observers could be more readily distracted by the high-SF information available through bottom-up processing. Accordingly, low-SF training could make observers more resistant to these changes when experiencing anxiety. Future work should seek to test these hypotheses empirically.

It is notable that RA was generally worse during the post and retention-tests for all groups than it was during the training itself, even when viewing the non-deceptive clips that were present during testing and training. We see two key differences that may help to explain the better performance during training. First, participants received feedback during training but not during testing, and the presence of feedback may have led to better performance during training. Second, the deceptive trials were mixed together with the non-deceptive trials in the post and retention tests (but absent during training), and therefore the uncertainty generated by the presence of the deceptive trials may have also reduced performance when viewing the non-deceptive trials (e.g., see Sarpeshkar et al., 2017). There has been growing interest not only in the ability of observers to exploit contextual information to *enhance* anticipatory performance (Abernethy et al., 2001; Cañal-Bruland and Mann, 2015), but also more recently on how the uncertainty generated by an increase in the number of likely outcomes can *decrease* performance (Mann et al., 2014; Sarpeshkar et al., 2017). Future work could look to examine how anticipatory performance changes in accordance with manipulations in the likelihood of a deceptive outcome, and whether blurred training aids in decreasing the degree to which observers are susceptible to the negative influences of contextual information.

It is worth considering whether our results might have been different if participants had viewed non-deceptive *and* deceptive actions during training. First, it seems reasonable to expect that the magnitude of the overall learning effect when compared from pre to post-test would have been greater, because participants would have become more accustomed to dealing with the uncertainty generated by the co-presentation of non-deceptive and deceptive clips (Sarpeshkar et al., 2017). When considering the low-SF training, because the low-SF clips remove the high-SF information that seemingly conveys deceptive intent (Abernethy et al., 2010a,b), then we would not expect any marked improvement in the ability of the low-SF group to anticipate deceptive actions on the basis of the kinematic information beyond that found in this study. However with the benefit of feedback, it could be that the high-SF group when training with both non-deceptive and deceptive actions would have learned which cues they could rely on to specify the actual motion outcome. Specifically, they could learn that the high-SF information is less specifying, and then rely on the low-SF information when it is available in the test. Given that this approach is likely to be quite explicit in nature, if true then we would still expect any gains as a result of high-SF training to be more likely to be lost when tested at retention as a result of ‘forgetting’ (Masters, 2008).

In this study we have employed a short-term intervention while training inexperienced observers to demonstrate a ‘proof-of-concept’ for the efficacy of low-SF training. Given the brief nature of the training (360 trials over 3 days), the magnitude of the increase in RA is reasonable (≈10–15%), with significant changes from pre to post-test supported by large effect sizes (*d*s > 0.8). The results do raise the question of whether the training would lead to similar improvements in the performance of more skilled observers (e.g., Hopwood et al., 2011). Skilled observers would be expected to already be more proficient in their ability to anticipate deceptive actions (Jackson et al., 2006), and so it is often considered to be more challenging to improve the already high anticipatory skill of better performers. Nonetheless, a concurrent study by van Biemen et al. (in review) has provided some suggestion that blurred perceptual training might also improve the decision making performance of *skilled* observers. In that study, evidence was found to suggest that the ability of skilled football referees to discriminate deceptive from non-deceptive actions (fouls vs. ‘dives’ in football) improved as a result of training when viewing blurred actions. Again, further work is warranted to determine the generalisability of these findings to a task where anticipation is required.

Given the recent concerns about the need for the testing and training of anticipation to be performed in conditions which accurately represent the performance environment (Mann et al., 2010a; Pinder et al., 2011; Abernethy et al., 2012; Mann and Savelsbergh, 2015), questions may naturally arise about the generalisability of our findings given that the task was performed when providing a button-press response while viewing video footage on a computer screen. In this study we were largely interested in examining the ability to anticipate deceptive intent, irrespective of whether it is performed by a person who must move to respond (e.g., a rugby defender) or rather must simply provide a perceptual response (e.g., a football referee). When interested in examining tasks where the observer would typically move, compromises are often made to maximize experimental control and convenience (Abernethy et al., 1993). In our case the compromise was largely borne out of necessity: because of the nature of the manipulations of SF, it would not have been possible to present the high-SF information that we used while viewing a live opponent. Manipulations which remove low-SF content act much like an ‘edge detector,’ and to our knowledge this was only possible using the manipulation of video footage. However, it is much simpler to perform low-SF training in the natural environment: participants can simply wear blurring glasses or contact lenses to achieve a similar effect (Applegate and Applegate, 1992; Mann et al., 2007, 2010b,c), making blur simpler and more applicable than point-light displays which are restricted for use with screen-based stimuli. Given the success of the low-SF training in this study, this now provides the opportunity to empirically (and practically) test the utility of low-SF training in the natural environment to establish whether our findings generalize to tasks where movements are required when responding to opponents *in situ*.

Finally, the findings from this study suggest that it may be possible to improve performance in other tasks where the perception of deception is crucial. Of course there are a range of scenarios from sports in which deception is vital, including one-on-one duals in rugby, tennis, baseball, and cricket. In each of those cases, successful transfer would rely on the findings from the present study, which were found when performing a perceptual task, to extend to tasks where perception and action are coupled. There certainly are though also *perceptual* tasks for which the perception of deception is vital. In addition to sport referees who are often required to discriminate genuine ‘fouls’ from situations in which an athletes ‘fakes’ a foul to gain a penalty (Renden et al., 2014), law enforcement officers or customs officials also often need to anticipate the actions of others (Cañal-Bruland, 2017). Another example is in Paralympic classification, where some athletes attempt to exaggerate their level of impairment to gain an advantage by being placed into a class designed for athletes with more severe impairment (Tweedy and Vanlandewijck, 2011; Ravensbergen et al., 2016; Tweedy et al., 2016; Mann and Ravensbergen, 2018). In these situations, the ability to ‘see through’ deceptive intent is vital, and low-SF training may hold promise as a means of improving the perception of deception if in those tasks success also relies on attunement to basic low-SF information.

## Conclusion

The findings of this study show that the ability to anticipate deceptive actions can be enhanced by training that removes superficial visual information. The outcomes support the idea that deceptive intent is underpinned by detailed high-SF information, and that attunement to low-SF visual information may prove to be a useful means for observers to become less-susceptible to the information that conveys deceptive intent.

## Ethics Statement

Ethical approval was obtained from the University of Hong Kong Human Research Ethics Committee prior to testing. All participants gave written informed consent in accordance with the Declaration of Helsinki.

## Author Contributions

DR, BA, and DM designed the study. DR and SP acquired the data. DR and DM contributed to the interpretation of the data and drafted the manuscript. BA revised the manuscript.

## Conflict of Interest Statement

The authors declare that the research was conducted in the absence of any commercial or financial relationships that could be construed as a potential conflict of interest.
